# Dr. W. T. G. Morton, of Boston

**Published:** 1860-05

**Authors:** 


DR. W. T. G. MORTON, OF BOSTON.—This gentleman has been in Philadelphia for some time past, endeavoring to induce the profession to recognize his claims as the discoverer of the anæsthetic effects of ether, and it affords us pleasure to state that his efforts have been completely successful, nearly all the respectable members signing the testimonial. The following preamble and resolutions were unanimously adopted at a meeting of the profession, on the twenty-sixth of March last, Dr. Wilson Jewell being in the chair, and Dr. T. H. Bache acting as secretary:

*Whereas*, after innumerable trials, made during the last fourteen years, it has been established to the satisfaction of the world, that the inhalation of ether may be safely employed for producing insensibility to pain;

*And whereas*, the attention of the medical profession, and through it of the public generally, was directed to this fact by Dr. Wm. T. G. Morton, of Boston, who first practically demonstrated that ether may be safely used by inhalation for annulling pain, in the Massachusetts General Hospital, where, on the 16th of October, 1846, a severe surgical operation was successfully performed by the late Dr. John C. Warren, without pain to the patient, while under the influence of ether, administered by Dr. Morton;

*And whereas*, our National Government, while admitting the claims of Dr. Morton, has failed to reward him for this great service to his country and to humanity:

*Resolved*, That in the opinion of this meeting, the world is indebted to Dr. Morton for having practically proved the value and safety of ether as an anæsthetic agent, and that he is, therefore, entitled to the lasting gratitude of mankind.

*Resolved*, That we cordially recommend to our fellow-citizens the "National Testimonial Fund," lately commenced in Boston and New York in behalf of Dr. Morton, and that we will do all in our power to influence them to subscribe to the same.

*Resolved*, That for the purpose of facilitating the subscriptions, a copy of these resolutions, duly authenticated by the officers of this meeting, be furnished to Messrs. Brown, Brothers & Co., the receivers for this city, together with such other documents in explanation of the Testimonial as may be approved by the officers of this meeting.

